# Development and Application of a Novel Machine Learning Model Predicting Pancreatic Cancer-Specific Mortality

**DOI:** 10.7759/cureus.57161

**Published:** 2024-03-29

**Authors:** Yongji Sun, Sien Hu, Xiawei Li, Yulian Wu

**Affiliations:** 1 Department of Surgery, Fourth Affiliated Hospital, Zhejiang University School of Medicine, Yiwu, CHN; 2 Department of Surgery, Second Affiliated Hospital, Zhejiang University School of Medicine, Hangzhou, CHN

**Keywords:** seer database, predictive model, survival quilts, machine learning, pancreatic ductal adenocarcinoma

## Abstract

Precise prognostication is vital for guiding treatment decisions in people diagnosed with pancreatic cancer. Existing models depend on predetermined variables, constraining their effectiveness. Our objective was to explore a novel machine learning approach to enhance a prognostic model for predicting pancreatic cancer-specific mortality and, subsequently, to assess its performance against Cox regression models. Datasets were retrospectively collected and analyzed for 9,752 patients diagnosed with pancreatic cancer and with surgery performed. The primary outcomes were the mortality of patients with pancreatic carcinoma at one year, three years, and five years. Model discrimination was assessed using the concordance index (C-index), and calibration was assessed using Brier scores. The Survival Quilts model was compared with Cox regression models in clinical use, and decision curve analysis was done. The Survival Quilts model demonstrated robust discrimination for one-year (C-index 0.729), three-year (C-index 0.693), and five-year (C-index 0.672) pancreatic cancer-specific mortality. In comparison to Cox models, the Survival Quilts models exhibited a higher C-index up to 32 months but displayed inferior performance after 33 months. A subgroup analysis was conducted, revealing that within the subset of individuals without metastasis, the Survival Quilts models showcased a significant advantage over the Cox models. In the cohort with metastatic pancreatic cancer, Survival Quilts outperformed the Cox model before 24 months but exhibited a weaker performance after 25 months. This study has developed and validated a novel machine learning-based Survival Quilts model to predict pancreatic cancer-specific mortality that outperforms the Cox regression model.

## Introduction

Pancreatic ductal adenocarcinoma (PDAC) stands as one of the major contributors to global cancer-related fatalities, and its incidence is on the rise [1]. Early detection poses a significant challenge, often occurring at an advanced stage. About 80% of patients receive a diagnosis when the disease has progressed substantially, resulting in limited long-term survival (2-9% at five years) [2]. While pancreatic resections at high-volume institutions show a relatively low mortality rate (3.8%), short-term morbidity remains high (30-40%) [3,4]. Thus, the imperative lies in refining patient selection and ensuring surgery benefits those likely to gain while sparing others from a potentially burdensome procedure with a low chance of long-term survival.

Past research has identified clinical and pathological factors predictive of overall survival in PDAC. For instance, Brennan et al. created a clinical nomogram based on 555 pancreatic resections, yielding a superior C-index of 0.64 in Cox multivariate analysis compared to traditional tumor, node, and metastasis (TNM) staging [5]. However, the clinical applicability is unsatisfactory, hampered by a modest sample size that compromises generalizability. Furthermore, existing personalized models rely heavily on traditional statistical methods with predetermined variables and interactions.

Amid the rapidly growing integration of machine learning in healthcare, novel prognostic models are emerging for various diseases [6-9]. Machine learning, a data-driven application of artificial intelligence, allows systems to autonomously learn and improve without explicit programming. Unlike traditional models, machine learning can explore datasets independently, identifying new variables and intricate relationships [10]. Recently, a novel machine learning-based approach produced a prognostic model called Survival Quilts to predict 10-year prostate cancer-specific mortality, which yielded similar performance to the top-ranked prognostic models using only standard clinicopathological variables [10]. However, this approach has not been applied to a large dataset for predicting short-term mortality in pancreatic cancer post-surgery.

Hence, this study aims to pioneer a novel machine learning approach to refine a prognostic model, Survival Quilts, for predicting pancreatic cancer-specific mortality. Additionally, it seeks to evaluate the performance of this approach against Cox regression models, providing a fresh perspective on prognostic modeling in the context of pancreatic cancer.

## Materials and methods

Data source

The patients diagnosed with pancreatic carcinoma in this study were specifically chosen from the Surveillance, Epidemiology, and End Results (SEER) "SEER Research Plus Data, 18 Registries, Nov 2020 Sub (2000-2018)" dataset, accessible at http://seer.cancer.gov. The SEER database encompasses information about cancer patients across 18 regions in the United States, representing approximately 28% of the entire national population. To identify patients with pancreatic cancer in the United States between 2010 and 2015, we utilized the SEER*Stat software version 8.4.0 (National Cancer Institute, Bethesda, Maryland, USA). Permission to access the database was granted through the completion and submission of the SEER Research Data Agreement form via email.

Study population

We identified patients diagnosed with pancreatic carcinoma by utilizing the site-recoded pancreas from the third revision of the International Classification of Diseases for Oncology codes (ICD-O-3). The inclusion criteria encompassed participants aged 20-80 years who were diagnosed with pancreatic carcinoma and underwent surgery between January 1, 2010, and December 31, 2015.

The exclusion criteria in this study were defined as follows: (a) patients with unknown survival months or survival months equal to zero, (b) patients with an unknown American Joint Committee on Cancer (AJCC) 7th TNM stage, (c) patients with an unknown histological grade, (d) patients with unknown tumor size, and (e) patients with unknown positive regional node status.

Predictors and outcome

Model inputs spanned a variety of demographic, clinical, and therapeutic variables. These covariates included in the analysis were age at diagnosis, sex, race, histologic grade, AJCC T category, AJCC N category, AJCC M category, regional nodes positive, CS tumor size, first malignant primary indicator, total number of in situ/malignant tumors, total number of benign/borderline tumors, primary site, histology recode-broad groupings, chemotherapy recode, and radiation recode. All variables were treated as categorical, except for age, tumor size, and regional nodes positive, which were considered continuous. The primary outcomes assessed were the mortality rates of patients diagnosed with pancreatic carcinoma at one year, three years, and five years.

Model development

We developed our machine learning-based survival model using Survival Quilts, an open-source software designed to automate the deployment of machine learning in survival analysis [11]. Survival Quilts, serving as an ensemble of diverse survival models, automatically weighs these models and adjusts their parameters in a single ensemble tailored to the specific dataset. The survival function generated by Survival Quilts combines profiles from various models, optimized to consider discriminative performance and calibration. As a result, Survival Quilts encompass multiple statistical and machine learning-based models for survival prediction. This automated software eliminates the need for researchers to choose a specific survival model, making machine learning expertise unnecessary. The study included four models, ranging from traditional statistical models to state-of-the-art deep learning models: Cox proportional hazards, random survival forest, conditional inference survival forest, and DeepHit models [12-14].

The SEER cohort was randomly split (8:2) into the training and testing sets using the Python version 3.9.0 package scikit-learn (Python Software Foundation, Wilmington, Delaware, USA). The training cohort is the portion of the dataset used to train the machine learning model. During the training process, the model learns patterns and relationships within the data. The test cohort is a separate portion of the dataset that is not used during the training phase but is reserved for evaluating the performance of the trained model. This cohort helps assess how well the model generalizes to new, unseen data. In this study, the training-cohort data were utilized to develop a Survival Quilts model, while the test-cohort data were employed to conduct Survival Quilts analysis, assessing the model's effectiveness in predicting the mortality of patients with pancreatic carcinoma. In parallel, multivariable Cox proportional hazards models were constructed using the same covariates for comparative analysis.

For model evaluation, the concordance index (C-index) was calculated to gauge model discrimination, and Brier scores were computed for calibration. Model calibration, which reflects the alignment between predicted and observed outcomes, was further evaluated through visual inspection of calibration plots.

The performance evaluation of both models commenced with an initial assessment of the entire cohort. Due to its critical impact on the prognosis of pancreatic cancer, including lymphatic and distant metastases, we conducted further analysis on two distinct groups: the non-metastatic pancreatic carcinoma population (individuals without lymphatic or distant metastasis, N0M0), and the metastatic pancreatic carcinoma population (individuals with lymphatic or distant metastasis).

Model visualization

We also developed a user-friendly interface to facilitate survival predictions. This interface consists of two views: the user input view and the mortality prediction view. The user input view is designed to help users input all entries regarding patient characteristics using the XML schema constructed based on the features input into the Survival Quilts model. The user input view allows users to predict mortality by clicking the predict button.

Statistical analysis

A two-sided p-value less than 0.05 was considered to be statistically significant. All statistical analyses were performed with SPSS Statistics version 25 (IBM Corp. Released 2017. IBM SPSS Statistics for Windows, Version 25.0. Armonk, NY: IBM Corp.) software. The C-index and Brier scores were calculated by Python version 3.9.0. All the figures were plotted by the Python (version 3.9.0) package matplotlib.

Role of funding source

All funding sources played no role in the design of the study, the collection, analysis, and interpretation of data, in writing the manuscript, or in the decision to submit the paper for publication.

## Results

Screening process

**Figure 1:**
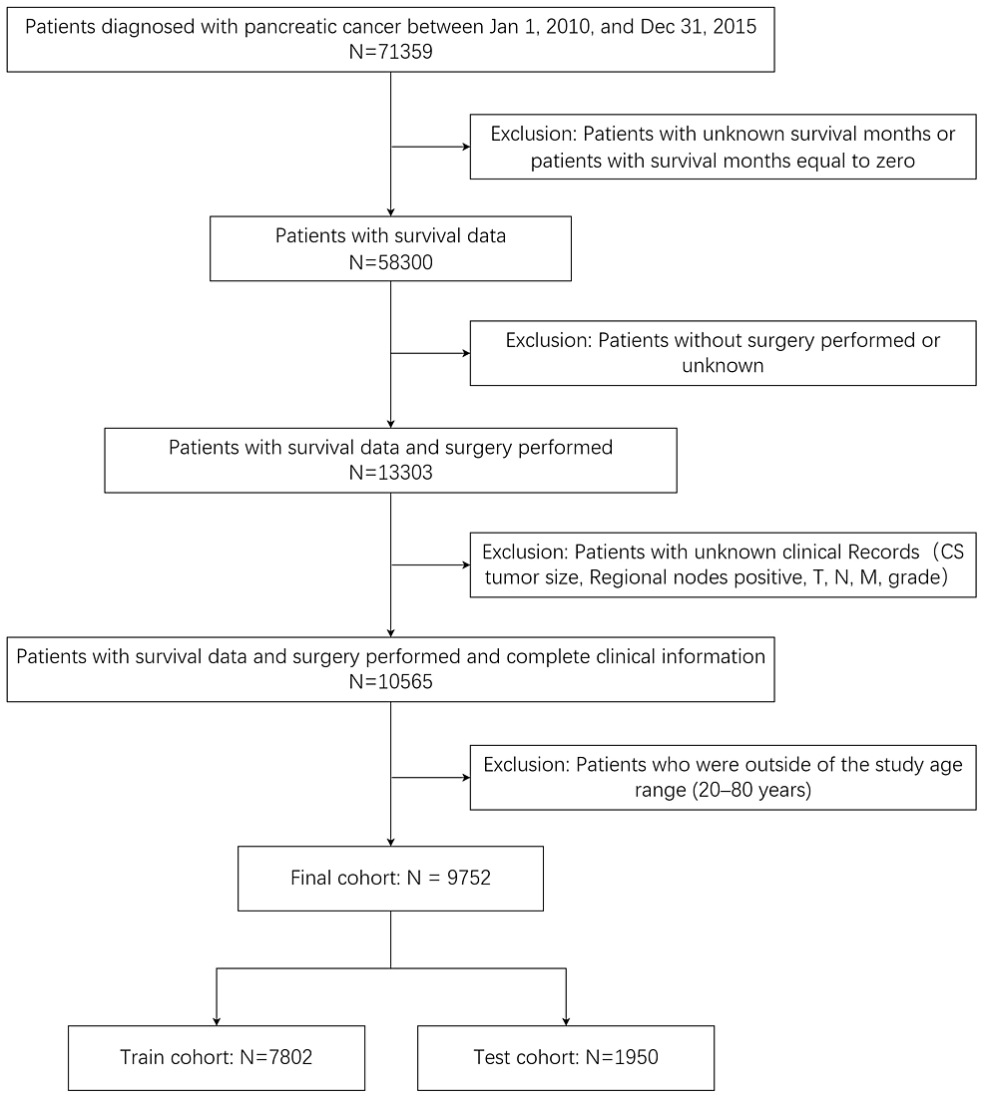
The flow diagram of patient data selection

Figure 1 illustrates our data assembly process. A total of 71,359 patients diagnosed with pancreatic cancer between January 1, 2010, and December 31, 2015, were enrolled in the SEER database. Among these, 13,059 patients lacked survival data or had survival months equal to zero, 44,997 patients did not undergo surgery or had unknown surgery status, and 2,738 patients had missing data for at least one essential domain (CS tumor size, regional node positive, TNM stage, grade). Additionally, 813 patients outside the study age range (20-80 years) were excluded. The final study population comprised 9,752 patients.

Baseline characteristics

Table 1 presents the main baseline clinical characteristics of the 9,752 patients with pancreatic carcinoma included in the study, comprising 4,726 female patients (51.5%) and 5,026 male patients (49.5%). The patients had an average age of 63.6 ± 10.5 years (mean ± SD), with the majority being of white ethnicity (80.7%). Nearly all cancers were categorized as T stage 1-3 (9,328 (95.7%)) and grade group 1-3 (9,594 (98.4%)). Lymph node metastases were evident in 5,840 patients (59.9%), while distant metastases were present in 614 patients (6.3%). The median (interquartile range) follow-up time stood at 27 (13-52) months, with 6,632 patients (68.0%) experiencing events during this period. Further details of the baseline information are available in Table 1.

**Table 1:** Demographic characteristics of patients included in the analysis AJCC: American Joint Committee on Cancer

Variables	Overall N=9752	Train cohort N=7802	Test cohort N=1950	p-value
					Survival months median (range)	27 (13-52)	27 (13-52)	27 (12-52)	0.856
Death status (%)				0.823
Alive	3120 (32.0)	2492 (31.9)	628 (32.2)	
Dead	6632 (68.0)	5310 (68.1)	1322 (67.8)	
Age at diagnosis mean±sd	63.6±10.5	63.7±10.5	63.4±10.4	0.13
Sex (%)				0.92
Female	4726 (48.5)	3779 (48.4)	947 (48.6)	
Male	5026 (51.5)	4023 (51.6)	1003 (51.4)	
Race recode (%)				0.663
White	7873 (80.7)	6296 (80.7)	1577 (80.9)	
Black	1004 (10.3)	797 (10.2)	207 (10.6)	
Others	875 (9.0)	709 (9.1)	166 (8.5)	
Grade (%)				0.559
Well differentiated; Grade I	2340 (24.0)	1867 (23.9)	473 (24.3)	
Moderately differentiated; Grade II	4330 (44.4)	3472 (44.5)	858 (44.0)	
Poorly differentiated; Grade III	2924 (30.0)	2330 (29.9)	594 (30.5)	
Undifferentiated; anaplastic; Grade IV	158 (1.6)	133 (1.7)	25 (1.3)	
Derived AJCC T, 7th ed (2010-2015) (%)				0.706
T1	1066 (10.9)	859 (11.0)	207 (10.6)	
T2	1427 (14.6)	1155 (14.8)	272 (13.9)	
T3	6835 (70.1)	5452 (69.9)	1383 (70.9)	
T4	424 (4.3)	336 (4.3)	88 (4.5)	
Derived AJCC N, 7th ed (2010-2015) (%)				0.787
N0	3912 (40.1)	3135 (40.2)	777 (39.8)	
N1	5840 (59.9)	4667 (59.8)	1173 (60.2)	
Derived AJCC M, 7th ed (2010-2015) (%)				0.898
M0	9138 (93.7)	7312 (93.7)	1826 (93.6)	
M1	614 (6.3)	490 (6.3)	124 (6.4)	
Primary site (%)				0.157
C25.0 head of pancreas	4944 (63.4)	1248 (64.0)	6192 (63.5)	
C25.1 body of pancreas	723 (9.3)	170 (8.7)	893 (9.2)	
C25.2 tail of pancreas	1340 (17.2)	307 (15.7)	1647 (16.9)	
Others	795 (10.2)	225 (11.5)	1020 (10.5)	
Histology recode - broad groupings (%)				0.589
Epithelial neoplasms, NOS	105 (1.3)	24 (1.2)	129 (1.3)	
Adenomas and adenocarcinomas	4451 (57.0)	1138 (58.4)	5589 (57.3)	
Cystic, mucinous, and serous neoplasms	321 (4.1)	88 (4.5)	409 (4.2)	
Ductal and lobular neoplasms	2790 (35.8)	665 (34.1)	3455 (35.4)	
Complex epithelial neoplasms	91 (1.2)	27 (1.4)	118 (1.2)	
Others	44 (0.6)	8 (0.4)	52 (0.5)	
Number of in situ/malignant tumors (%)				0.128
1	5938 (76.1)	1516 (77.7)	7454 (76.4)	
>1	1864 (23.9)	434 (22.3)	2298 (23.6)	
Number of benign/borderline tumors (%)				0.803
0	7750 (99.3)	1938 (99.4)	9688 (99.3)	
>0	52 (0.7)	12 (0.6)	64 (0.7)	
First malignant primary indicator (%)				0.232
No	1705 (17.5)	1382 (17.7)	323 (16.6)	
Yes	8047 (82.5)	6420 (82.3)	1627 (83.4)	
Regional node positive median (range)	1 (0-3）	1 (0-3）	1 (0-3）	0.944
CS tumor size median (range), mm	31 (23-43)	31 (23-43)	30 (23-43)	0.544
Radiotherapy (%)				0.358
No/unknown	5714 (73.2)	1408 (72.2)	7122 (73.0)	
Yes	2088 (26.8)	542 (27.8)	2630 (27.0)	
Chemotherapy (%)				0.858
No/unknown	2998 (38.4)	745 (38.2)	3743 (38.4)	
Yes	4804 (61.6)	1205 (61.8)	6009 (61.6)	

Additionally, demographic characteristics in the two study cohorts (train cohort and test cohort) are presented in Table 1, demonstrating no significant differences in clinical characteristics between the cohorts (p>0.05).

Model performance in the full cohort

The predictive performance of the models was measured using the C-index, Brier score, calibration plots, and decision curve analysis. The values of the C-index and Brier score for each model are presented in Table 2.

**Table 2:** Discrimination and calibration of each model at predicting one-, three-, and five-year mortality

	C-index	Brier score
1 year		
Survival Quilts	0.726	0.211
Cox	0.698	0.211
3 year		
Survival Quilts	0.693	0.299
Cox	0.698	0.299
5 year		
Survival Quilts	0.672	0.244
Cox	0.695	0.232

**Figure 2:**
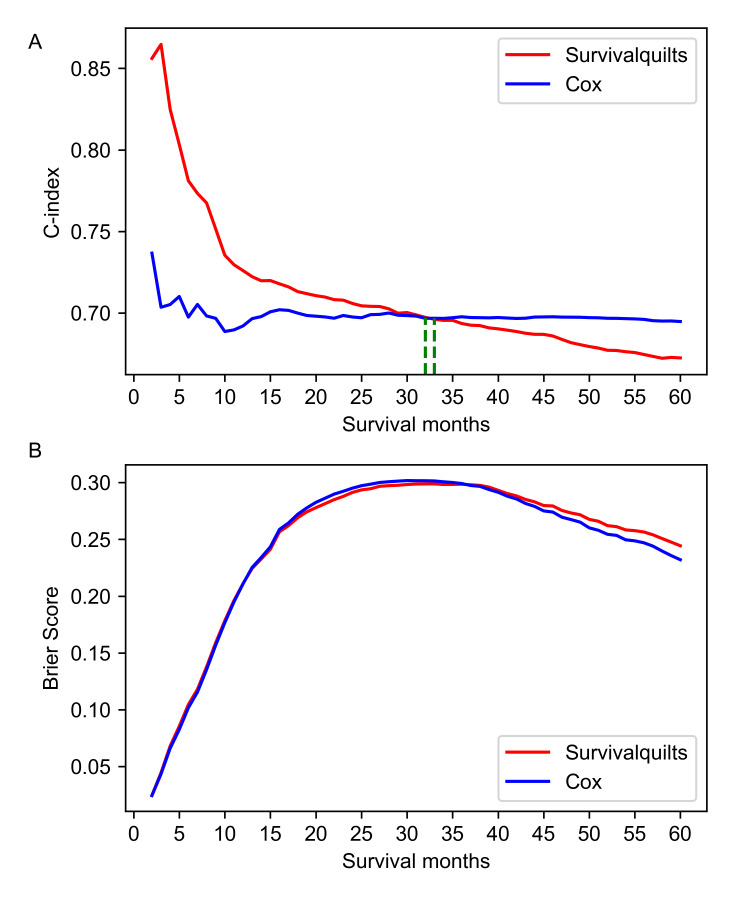
Overtime C-index and Brier score for predicting pancreatic cancer-specific mortality The evaluation of predictive performance for pancreatic cancer-specific mortality involves the consideration of the overtime C-index (denoted as A) and Brier score (represented by B).

For the one-year mortality prediction, the Survival Quilts model (C-index 0.726) showed better discrimination than the Cox model (C-index 0.698). However, for the three- and five-year mortality prediction, the C-index of the Survival Quilts model (thee-year: 0.693; five-year: 0.672) was lower than that of the Cox model (three-year: 0.698; five-year:0.695). The overtime C-index in Figure 2 revealed that the Survival Quilts model exhibited a higher C-index before 32 months but performed worse after 33 months.

**Figure 3:**
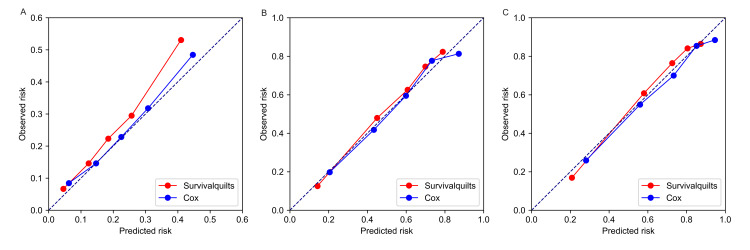
Calibration plots of observed versus predicted risk. The prediction of pancreatic cancer-specific mortality at one-year (denoted as A), three-year (denoted as B), and five-year (denoted as C) intervals utilized Survival Quilts and Cox models.

**Figure 4:**
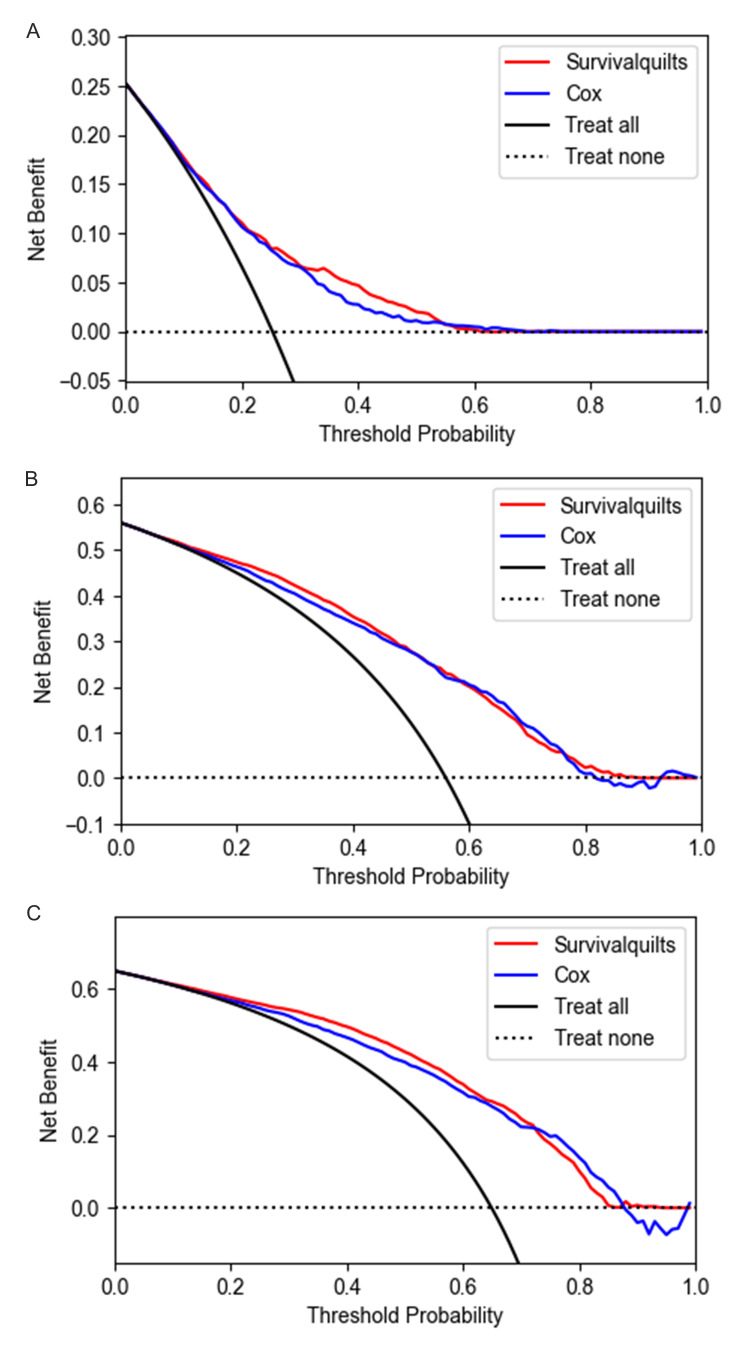
Decision curve analysis The prediction of pancreatic cancer-specific mortality at one-year (denoted as A), three-year (denoted as B), and five-year (denoted as C) intervals utilized Survival Quilts and Cox models. (The clinical net benefit for each prediction model is calculated across a range of risk threshold probabilities. Clinical net benefit is defined as the minimum probability of disease at which further intervention would be warranted.)

The model's calibration was assessed using the Brier score and calibration plots. The overtime Brier score plots in Figure 2 showed no significant difference between the two models before 57 months (the difference being less than 0.01), with the Cox model performing slightly better after 57 months (the difference being more than 0.01). Regarding the calibration plot (Figure 3), actual outcomes for three- and five-years were highly consistent with predictions from both models, with most points closely aligning with the 45° line. However, in the Survival Quilts subsequently, we evaluated model performance through decision curve analysis, considering the impact on treatment decision-making. For the one-year prediction, the Survival Quilts model provided a greater gain than the Cox model, although there was no significant difference between the two models in predicting three- and five-year mortality (Figure 4).

Model performance in subgroups

**Figure 5:**
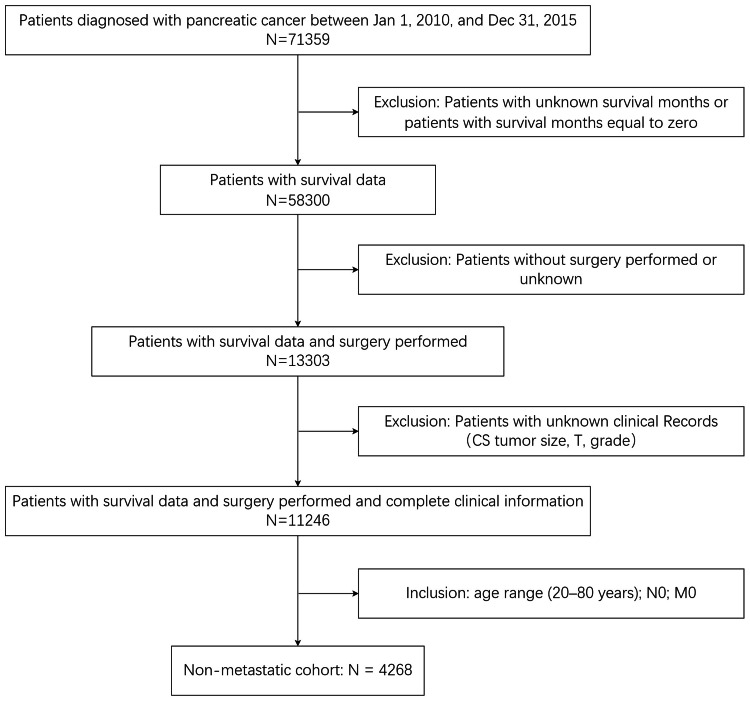
The flow diagram of patients with non-metastatic pancreatic carcinoma selection

**Figure 6:**
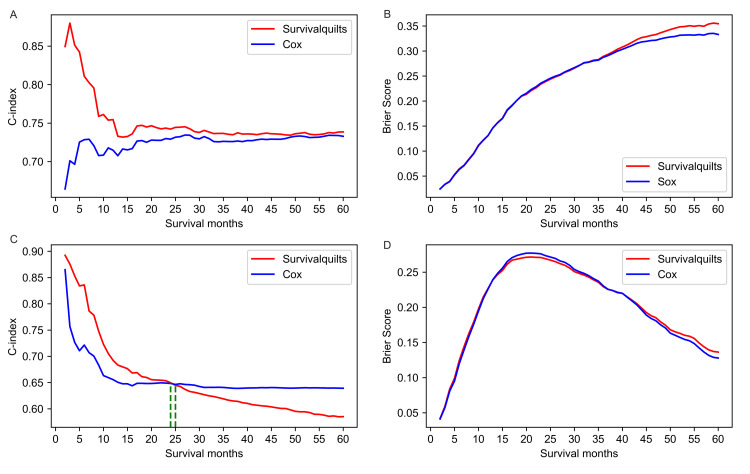
The evaluation of predictive performance for the mortality of both non-metastatic and metastatic pancreatic cancer (A) Overtime C-index for predicting mortality in non-metastatic pancreatic cancer. (B) Overtime Brier score for predicting mortality in non-metastatic pancreatic cancer. (C) Overtime C-index for predicting mortality in metastatic pancreatic cancer. (D) Overtime Brier score for predicting mortality in metastatic pancreatic cancer.

**Figure 7:**
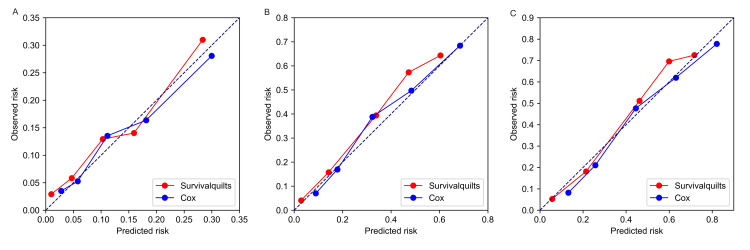
Calibration plots of the non-metastatic cohort The prediction of non-metastatic pancreatic cancer-specific mortality at one-year (denoted as A), three-year (denoted as B), and five-year (denoted as C) intervals utilized Survival Quilts and Cox models.

**Figure 8:**
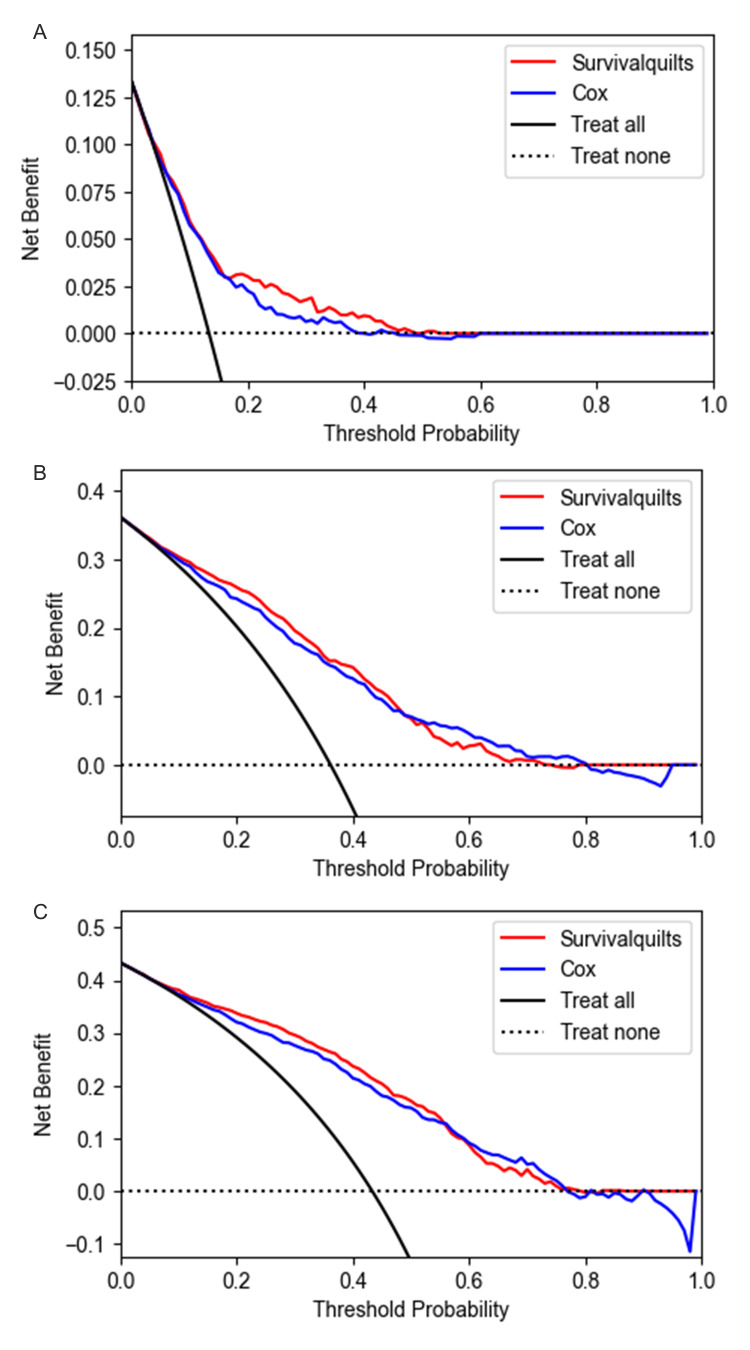
Decision curve analysis of the non-metastatic cohort The prediction of non-metastatic pancreatic cancer-specific mortality at one-year (denoted as A), three-year (denoted as B), and five-year (denoted as C) intervals utilized Survival Quilts and Cox models.

We initially focused on the population with non-metastatic pancreatic carcinoma. The screening process and baseline characteristics are detailed in Figure 5 and Table 3. Overtime C-index and Brier scores are depicted in Figure 6. Concerning model discrimination, the Survival Quilts models consistently outperformed the Cox models, displaying a higher C-index from two to 60 months. In terms of model calibration, no significant differences were observed before 45 months, but the Survival Quilts model exhibited slightly poorer performance after 45 months. Calibration plots and decision curve analyses for one-, three-, and five-year mortality predictions are included in Figures 7-8.

**Table 3:** Demographic characteristics of patients with non-metastatic pancreatic cancer AJCC: American Joint Committee on Cancer

Variables	Overall N=4268	Train cohort N=3414	Test cohort N=854	p-value
					Survival months median (range)	44 (21-65)	44 (21-65）	44 (20-64）	0.471
Death status (%)				0.976
Alive	2287 (53.6)	1829 (53.6)	458 (53.6)	
Dead	1981 (46.4)	1585 (46.4)	396 (46.4)	
Age at diagnosis mean±sd	62.8±11.3	62.7±11.4	63.1±10.9	0.443
Sex (%)				0.277
Female	2145 (50.3)	1730 (50.7)	415 (48.6)	
Male	2123 (49.7)	1684 (49.3)	439 (51.4)	
Race recode (%)				0.208
White	3373 (79.0)	2712 (79.4)	661 (77.4)	
Black	432 (10.1)	346 (10.1)	86 (10.1)	
Others	463 (10.8)	356 (10.4)	107 (12.5)	
Grade (%)				0.921
Well differentiated; Grade I	1749 (41.0)	1395 (40.9)	354 (41.5)	
Moderately differentiated; Grade II	1638 (38.4)	1315 (38.5)	323 (37.8)	
Poorly differentiated; Grade III	812 (19.0)	647 (19.0)	165 (19.3)	
Undifferentiated; anaplastic; Grade IV	69 (1.6)	57 (1.7)	12 (1.4)	
Derived AJCC T, 7th ed (2010-2015) (%)				0.981
T1	1159 (27.2)	929 (27.2)	230 (26.9)	
T2	1015 (23.8)	813 (23.8)	202 (23.7)	
T3	1958 (45.9)	1565 (45.8)	393 (46.0)	
T4	136 (3.2)	107 (3.1)	29 (3.4)	
Primary site (%)				0.572
C25.0 head of pancreas	2051 (48.1)	1623 (47.5)	428 (50.1)	
C25.1 body of pancreas	587 (13.8)	476 (13.9)	111 (13.0)	
C25.2 tail of pancreas	1072 (25.1)	862 (25.2)	210 (24.6)	
Others	558 (13.1)	453 (13.3)	105 (12.3)	
Histology recode - broad groupings (%)				0.922
Epithelial neoplasms, NOS	62 (1.5)	53 (1.6)	9 (1.1)	
Adenomas and adenocarcinomas	2850 (66.8)	2280 (66.8)	570 (66.7)	
Cystic, mucinous, and serous neoplasms	232 (5.4)	184 (5.4)	48 (5.6)	
Ductal and lobular neoplasms	1050 (24.6)	839 (24.6)	211 (24.7)	
Complex epithelial neoplasms	49 (1.1)	38 (1.1)	11 (1.3)	
Others	25 (0.6)	20 (0.6)	5 (0.6)	
Number of in situ/malignant tumors (%)				0.938
1	3133 (73.4)	2507 (73.4)	626 (73.3)	
>1	1135 (26.6)	907 (26.6)	228 (26.7)	
Number of benign/borderline tumors (%)				0.426
0	4228 (99.1)	3380 (99.0)	848 (99.3)	
>0	40 (0.9)	34 (1.0)	6 (0.7)	
First malignant primary indicator (%)				0.499
No	810 (19.0)	641 (18.8)	169 (19.8)	
Yes	3458 (81.0)	2773 (81.2)	685 (80.2)	
CS tumor size median (range), mm	25 (17-39)	25 (17-39)	27(18-39)	0.385
Radiotherapy (%)				0.462
No/unknown	3461 (81.1)	2776 (81.3)	685 (80.2)	
Yes	807 (18.9)	638 (18.7)	169 (19.8)	
Chemotherapy (%)				
No/unknown	2388 (56.0)	1916 (56.1)	472 (55.3)	0.654
Yes	1880 (44.0)	1498 (43.9)	382 (44.7)	

**Figure 9:**
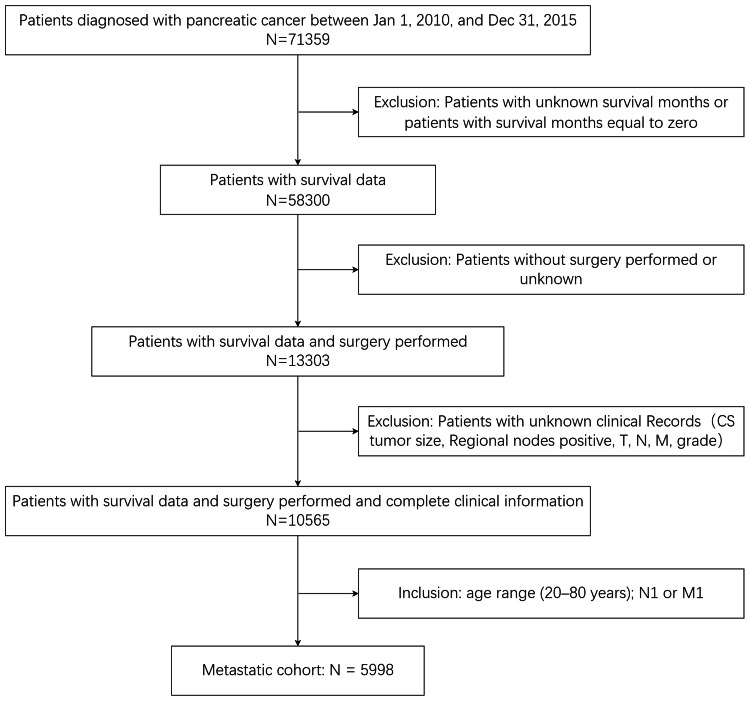
The flow diagram of patients with metastatic pancreatic carcinoma selection

**Figure 10:**
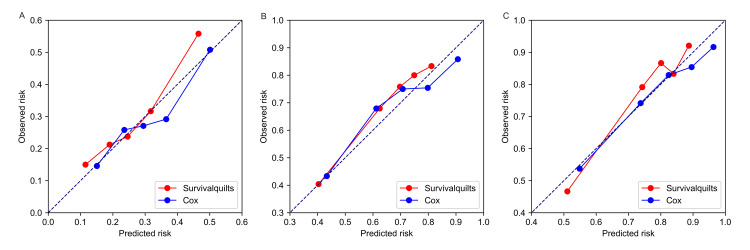
Calibration plots of the metastatic cohort The prediction of metastatic pancreatic cancer-specific mortality at one-year (denoted as A), three-year (denoted as B), and five-year (denoted as C) intervals utilized Survival Quilts and Cox models.

**Figure 11:**
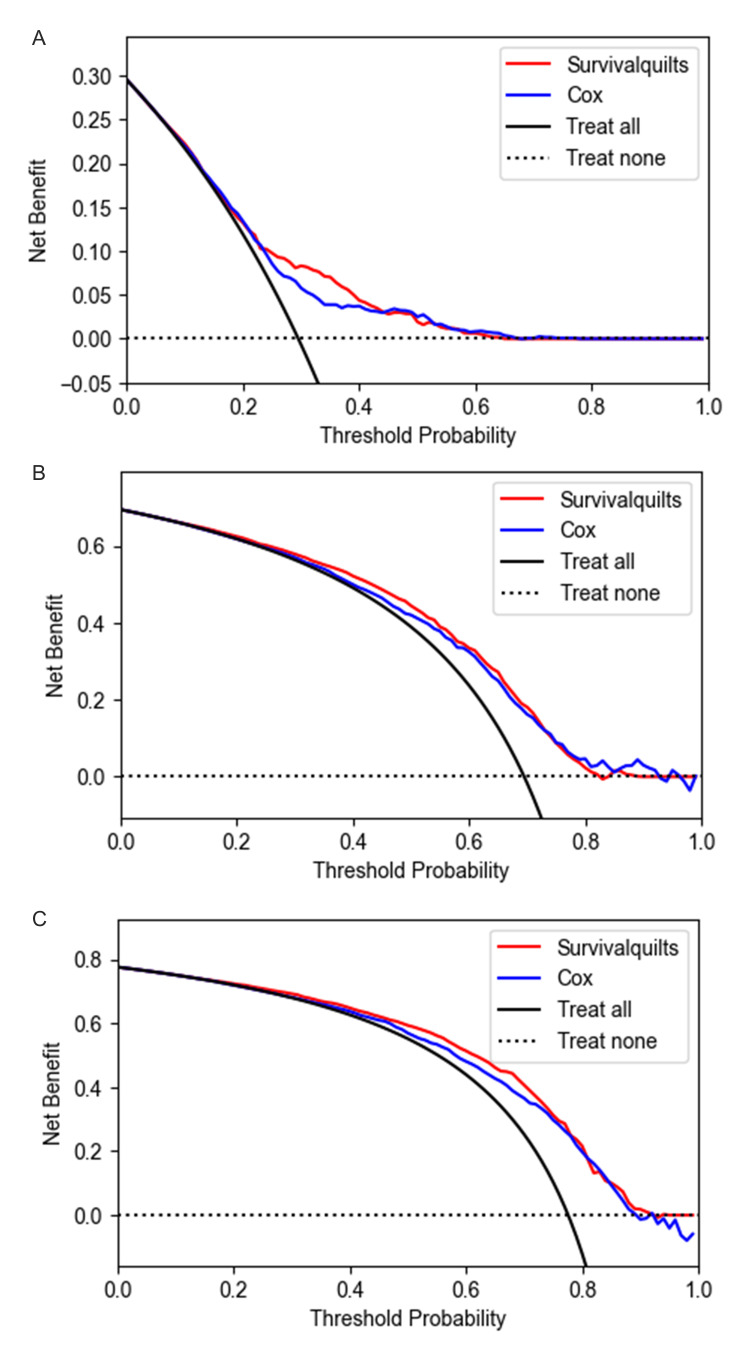
Decision curve analysis of the metastatic cohort The prediction of metastatic pancreatic cancer-specific mortality at one-year (denoted as A), three-year (denoted as B), and five-year (denoted as C) intervals utilized Survival Quilts and Cox models.

Subsequently, we explored the performance of the Survival Quilts model in the cohort with metastatic pancreatic carcinoma. The screening process and baseline characteristics for this cohort are outlined in Figure 9 and Table 4. Overtime C-index and Brier scores are presented in Figure 6. The findings mirror those of the full cohort, where the Survival Quilts model demonstrated a higher C-index before 24 months but performed less effectively after 25 months. Regarding the Brier score, no significant differences were observed between the two models from two to 60 months. Calibration plots and decision curve analyses for one-, three-, and five-year mortality predictions are provided in Figures 10-11.

**Table 4:** Demographic characteristics of patients with metastatic pancreatic cancer AJCC: American Joint Committee on Cancer

Variables	Overall N=5998	Train cohort N=4798	Test cohort N=1200	p-value
					Survival months median (range)	20 (11-41)	20 (10-41)	21 (11-41)	0.93
Death status (%)				0.903
Alive	1212 (20.2)	968 (20.2)	244 (20.3)	
Dead	4786 (79.8)	3830 (79.8)	956 (79.7)	
Age at diagnosis mean±sd	64.0±10.0	64.0±9.9	63.9±10.2	0.956
Sex (%)				0.422
Female	2842 (47.4)	2261 (47.1)	581 (48.4)	
Male	3156 (52.6)	2537 (52.9)	619 (51.6)	
Race recode (%)				0.763
White	4907 (81.8)	3930 (81.9)	977 (81.4)	
Black	607 (10.1)	487 (10.2)	120 (10.0)	
Others	484 (8.1)	381 (7.9)	103 (8.6)	
Grade (%)				0.865
Well differentiated; Grade I	935 (15.6)	744 (15.5)	191 (15.9)	
Moderately differentiated; Grade II	2818 (47.0)	2263 (47.2)	555 (46.3)	
Poorly differentiated; Grade III	2149 (35.8)	1712 (35.7)	437 (36.4)	
Undifferentiated; anaplastic; Grade IV	96 (1.6)	79 (1.6)	17 (1.4)	
Derived AJCC T, 7th ed (2010-2015) (%)				0.359
T1	161 (2.7)	133 (2.8)	28 (2.3)	
T2	564 (9.4)	437 (9.1)	127 (10.6)	
T3	4965 (82.8)	3984 (83.0)	981 (81.8)	
T4	308 (5.1)	244 (5.1)	64 (5.3)	
Derived AJCC N, 7th ed (2010-2015) (%)				0.258
N0	158 (2.6)	132 (2.8)	26 (2.2)	
N1	5840 (97.4)	4666 (97.2)	1174 (97.8)	
Derived AJCC M, 7th ed (2010-2015) (%)				0.466
M0	5384 (89.8)	4300 (89.6)	1084 (90.3)	
M1	614 (10.2)	498 (10.4)	116 (9.7)	
Primary site (%)				0.566
C25.0 head of pancreas	4279 (71.3)	3407 (71.0)	872 (72.7)	
C25.1 body of pancreas	406 (6.8)	332 (6.9)	74 (6.2)	
C25.2 tail of pancreas	755 (12.6)	614 (12.8)	141 (11.8)	
Others	558(9.3)	445(9.3)	113(9.4)	
Histology recode - broad groupings (%)				0.615
Epithelial neoplasms, NOS	74(1.2)	62(1.3)	12(1.0)	
Adenomas and adenocarcinomas	3204(53.4)	2578(53.7)	626(52.2)	
Cystic, mucinous, and serous neoplasms	187(3.1)	146(3.0)	41(3.4)	
Ductal and lobular neoplasms	2429 (40.5)	1929 (40.2)	500 (41.7)	
Complex epithelial neoplasms	71 (1.2)	59 (1.2)	12 (1.0)	
Others	33 (0.6)	24 (0.5)	9 (0.8)	
Number of in situ/malignant tumors (%)				0.116
1	4693 (78.2)	3734 (77.8)	959 (79.9)	
>1	1305 (21.8)	1064 (22.2)	241 (20.1)	
Number of benign/borderline tumors (%)				0.859
0	5966 (99.5)	4772 (99.5)	1194 (99.5)	
>0	32 (0.5)	26 (0.5)	6 (0.5)	
First malignant primary indicator (%)				0.599
No	1000 (16.7)	806 (16.8)	194 (16.2)	
Yes	4998 (83.3)	3992 (83.2)	1006 (83.8)	
Regional node positive median (range)	3 (1-5)	3 (1-5)	3 (1-5)	0.441
CS tumor size median(range), mm	35 (25-45)	35 (25-45)	34 (25-45)	0.19
Radiotherapy (%)				0.262
No/unknown	4134 (68.9)	3323 (69.3)	811 (67.6)	
Yes	1864 (31.1)	1475 (30.7)	389 (32.4)	
Chemotherapy (%)				0.346
No/unknown	1776 (29.6)	1434 (29.9)	342 (28.5)	
Yes	4222 (70.4)	3364 (70.1)	858 (71.5)	

Model visualization

**Figure 12:**
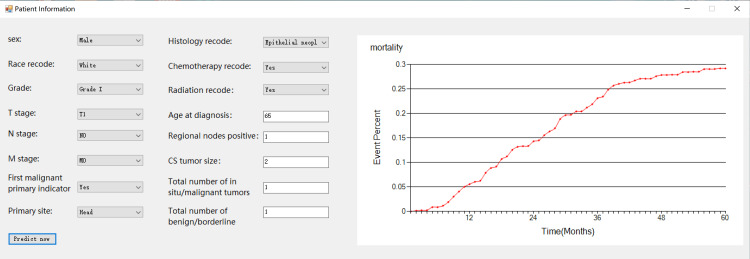
User-friendly interface of the Survival Quilts model

In the prediction view, the system invokes a prediction model, as shown in Figure 12, and the Survival Quilts model is used to predict patients’ mortality. The analysis results are visualized in a graphic view as a mortality curve, which indicates the mortality of the patient input overtime.

## Discussion

In this investigation, we utilized an extensive dataset to formulate and evaluate an innovative prognostic model, trained through machine learning, designed for the prediction of pancreatic cancer-specific mortality. Its performance was systematically compared against traditional Cox regression models. To the best of our knowledge, our research represents the pioneering application of Survival Quilts for the prediction of pancreatic cancer-specific mortality. Our findings indicate that Survival Quilts demonstrated superior predictive accuracy compared to the Cox model for short-term mortality in postoperative pancreatic cancer patients. However, its efficacy lagged behind the Cox model in forecasting long-term mortality. This study introduces a pioneering approach to predicting pancreatic cancer mortality, leveraging a novel machine learning algorithm that automatically amalgamates optimal attributes derived from diverse modeling methods.

Pancreatic cancer, characterized by a dismal prognosis, witnessed a nearly 45% mortality rate within two years for 4,416 out of 9,752 individuals in our cohort. Therefore, the prediction of short-term mortality in pancreatic cancer holds more substantial clinical importance than long-term mortality. Our Survival Quilts model exhibits significantly superior performance in predicting mortality within two years compared to the efficiency of Cox. Subgroup analysis within the pancreatic cancer population revealed a notable advantage of the Survival Quilts models over the Cox models among individuals without metastasis. In the metastatic pancreatic cancer population, Survival Quilts outperformed the Cox model in short-term mortality prediction but showed reduced efficacy in predicting long-term mortality. Within this cohort, 30% of patients succumbed within one year (1,839/5,998) and 55% within two years (3,353/5,998), underscoring the heightened clinical significance of short-term mortality prediction. Additionally, our Survival Quilts model consistently demonstrated a significant advantage over the Cox model in predicting mortality within two years. We conclude that, for forecasting the risk of pancreatic cancer mortality, Survival Quilts exhibits superior clinical utility compared to Cox.

Several studies employing machine learning techniques for pancreatic cancer prognostics exist, though many of them have employed small cohorts during model development. For instance, Keyl et al. [15], in a study involving 203 pancreatic cancer patients, formulated a prognostic model utilizing a random survival forest based on clinical indicators. They achieved a c-index of 0.71, significantly surpassing survival risk prediction based on the AJCC system. Owing to the low incidence of pancreatic cancer [16], the limited sample size has impeded the development of reliable machine learning models in previous research. In contrast, our investigation, leveraging the SEER database, encompassed a substantial total of 9,752 postoperative samples of pancreatic cancer. This ample sample size created favorable conditions for the robust development of machine learning models.

Beyond sample size considerations, the selection of an appropriate machine learning method is paramount for constructing a dependable prognostic model. Previous studies often employed a strategy of simultaneously applying diverse machine learning methods and selecting the most efficient algorithm as the final model [17,18]. For instance, in a study involving a total of 1,280 pancreatic cancer samples from multiple centers, Wang et al. [17] employed 76 different machine learning algorithms to construct models, ultimately opting for CoxBoost and Survival Support Vector Machine as the final prognostic models. Our study introduces a novel machine learning method, Survival Quilts, in the construction of the prognostic model. Survival Quilts, operating as an ensemble of diverse survival models, automatically assigns weights to these models and adjusts their parameters in a single ensemble tailored to the specific dataset. In essence, Survival Quilts automates the process of optimal model selection.

Our machine learning model was developed through training on a sizable and diverse contemporary population, utilizing real-world data from a robust database. As far as we know, this study represents the most extensive application of machine learning in pancreatic cancer prognostics. Nevertheless, it is essential to acknowledge some significant limitations in this study. First, due to the absence of comorbidity and treatment data in the SEER database, we were unable to incorporate these crucial variables into our modeling. This limitation precluded our ability to assess the impact of comorbidity on outcomes and consider the effects of treatment, factors that play pivotal roles in other predictive models. Second, SEER does not capture information on CT or molecular markers, which are instrumental in predicting the prognosis of pancreatic cancer [19]. Additionally, our study lacks external validation, highlighting the need for further investigation to substantiate the benefits of employing Survival Quilts in the realm of survival prediction.

## Conclusions

We have developed and validated a novel machine learning-based Survival Quilts model to predict pancreatic cancer-specific mortality, which outperforms the Cox regression model. A user-friendly interface has also been built for clinical applications. The future incorporation of supplementary data is expected to enhance the performance and accuracy of the model, providing better personalized prognostic outcomes.
